# Towards Adaptive Information Visualization - A Study of Information Visualization Aids and the Role of User Cognitive Style

**DOI:** 10.3389/frai.2019.00022

**Published:** 2019-11-01

**Authors:** Ben Steichen, Bo Fu

**Affiliations:** ^1^Department of Computer Science, California State Polytechnic University, Pomona, CA, United States; ^2^Department of Computer Engineering and Computer Science, California State University, Long Beach, CA, United States

**Keywords:** information visualization, adaptation, cognitive style, interaction, human-centered computing, personalization

## Abstract

Information Visualization systems have traditionally followed a one-size-fits-all model, whereby the same visualization is shown to each user, without taking into consideration an individual user's preferences, abilities, or context. By contrast, given the considerable cognitive effort involved in using Information Visualizations, this paper investigates the effect of an individual user's cognitive style on Information Visualization performance. In addition, this paper studies several interactive “visualization aids” (i.e., interactive overlays that can aid in visualization comprehension), as well as the effect of cognitive style on aid choices and preferences. The results from a user study show that cognitive style plays a significant role when performing tasks with Information Visualizations in general, and that there are clear differences in terms of individual aid choices and preferences. These findings also provide motivation for the development of adaptive and personalized Information Visualization systems that could better assist users according to their individual cognitive style.

## Introduction

One of the most powerful ways to help humans perform cognitive work is to support them with interactive visualizations, particularly through computer-generated Information Visualizations (Spence, 2001; Ware, 2004). Given the unprecedented amount of information now available to people, organizations, and communities, the use of Information Visualization systems has become ubiquitous for diverse populations across a wide variety of activities, such as reading newspaper articles, exploring scientific data, or making business decisions.

Traditionally, Information Visualization systems have followed a one-size-fits-all model, whereby the same (often non-interactive) visualization is shown to each user, without taking into consideration an individual user's preferences, abilities, or context. By contrast, in fields outside of Information Visualization, there are ample established examples of successfully designing systems that are personalized to individual users, such as in Personalized Information Retrieval (Steichen et al., 2012), Adaptive Web systems (Steichen et al., 2012), or Adaptive E-learning (Jameson, 2008).

In the field of Information Visualization, such research regarding interaction, adaptation, and personalization has emerged only recently, showing that individual user characteristics may have an impact on Information Visualization effectiveness, and that there is potential for the development of adaptive and personalized Information Visualization solutions. As with any development of such systems, researchers have focused on (i) determining what specific characteristics may play a role in a user's interaction with a system, and (ii) devising mechanisms to help users.

In this paper, we similarly focus on both of these aspects, and extend prior work by (i) investigating the effect that a user's cognitive style may have on their use of Information Visualizations, and (ii) investigating several general Information Visualization “aids” that may be added to an existing visualization to assist users during typical tasks (i.e., interactive overlays that can aid in visualization comprehension).

The focus on a user's cognitive style is based on several related research works outside of Information Visualization, which have shown that this user characteristic can have significant effects on a user's processing of visual information (Witkin et al., 1975; Mawad et al., 2015; Raptis et al., 2016). Since using Information Visualizations consists of complex cognitive activities that make significant use of visual information, we hypothesize that this characteristic may therefore have a significant impact.

The focus on general visualization “aids” that are added to an existing visualization (i.e., visualization overlays) is motivated by the fact that prior work has so far mostly concentrated on visualization highlighting effects (Carenini et al., 2014) (i.e., highlighting specific data points), which by definition require the system to know exactly which data points the user is most interested in. While this is a valid assumption in the case of systems that, for example, present visualizations along with a textual description (e.g., a newspaper article that is accompanied by a visualization), this is not generally the case. In particular, users may be engaged in several different tasks on a single visualization, and the visualization system developer/provider may not know which aspects or data points the user is focused on at any given time. More general Information Visualization aids, such as grid overlays or added labels, may therefore be more appropriate in such cases.

In order to investigate these Information Visualization aids, as well as the role of a user's cognitive style, this paper presents a user study where participants interacted with two common Information Visualizations, namely bar graphs and line graphs, and five different visualization aids. The specific research questions that this user study aims to answer are:

1. To what extent does a user's cognitive style play a role when performing tasks with Information Visualization systems? (RQ1)
2. In general, which Information Visualization Aids do users choose the most, and which are considered most helpful by users? (RQ2)
3. Does cognitive style play a role in aid choice and subjective usefulness? (RQ3).

## Related Work

Research on the effect of, and adaptation to, individual user characteristics has long been established in fields outside of Information Visualization. Prominent examples include Adaptive Hypermedia (Steichen et al., 2012), Personalized Information Retrieval (Steichen et al., 2012), and Adaptive e-Learning (Jameson, 2008). In each of these fields, the first step is to identify an influential user characteristic, followed by research on how to best support each individual user in a personalized manner. For example, the goal of many Personalized Information Retrieval systems is to personally tailor search results to each individual user (Steichen et al., 2012). In order to achieve this goal, systems may employ a range of techniques to, for example, (i) gather individual user interests from prior queries and result selections, in order to (ii) tailor retrieval algorithms to re-rank search results based on these interests. Likewise, Adaptive e-Learning systems may (i) gather a user's knowledge through tests or interaction patterns, in order to (ii) provide a personalized path through the learning material.

### Human Factors and Information Visualization

Besides the above examples of “traditional” user characteristics (e.g., user interests or prior knowledge), more recent work has also investigated the effect of human factors, such as cognitive processing capabilities (Germanakos et al., 2009). In particular, one human factor that has been consistently shown to influence human behavior is the high-level cognitive process of cognitive style. Specifically, according to the (FD-I) theory, *Field Dependent* people tend to have difficulties in identifying details in complex scenes, whereas *Field Independent* people easily separate structures from surrounding visual context (Witkin et al., 1975). This characteristic has been shown to have significant effects in several areas outside of Information Visualization, for example when playing games (Raptis et al., 2016) or making purchasing decisions (Mawad et al., 2015). Specifically, gamers have been shown to have varying completion speeds and behavioral patterns depending on this characteristic (Raptis et al., 2016). Likewise, users showed different information processing behaviors when reading product labels (Mawad et al., 2015). Recent research has shown that such differences can even be implied from eye gaze data (Mawad et al., 2015; Raptis et al., 2017). Given the intricate connection of this user characteristic with visual tasks, our paper therefore hypothesizes that it may also have an influence on Information Visualization use.

The effect of individual user differences and human factors on behaviors with Information Visualizations has only been studied very recently. Most notably, there are a number of examples showing that there is an effect of personality, cognitive abilities, and expertise on a user's performance with (and preference for) different visualizations (Velez et al., 2005; Green and Fisher, 2010; Ziemkiewicz et al., 2011; Toker et al., 2012; Carenini et al., 2014; Luo, 2019). For example, results in Ziemkiewicz et al. (2011) showed that users with an internal locus of control performed poorly with Information Visualizations that employ a containment metaphor, while those with an external locus of control showed good performance with such systems. This finding provided motivation for the tailoring/selection of different Information Visualizations for different users, depending on their locus of control. Similarly, results in Toker et al. (2012) showed that user cognitive abilities, such as perceptual speed and working memory had an influence on visualization preferences and task completion time. Most recently, Luo (2019) investigated user cognitive style along the visualizer-verbalizer dimension (Richardson, 1977; Riding, 2001), where individuals were distinguished as either preferring their visual or verbal subsystem. Based on this distinction, results showed that verbalizers preferred table representations of data, whereas visualizers preferred graphical representations (i.e., data visualizations). However, the effects of a user's cognitive style according to the (FD-I) theory have, to the best of our knowledge, not been explored in Information Visualization, despite its proven effect on visual tasks in other fields (Mawad et al., 2015; Raptis et al., 2016, 2017). Our paper addresses this research gap by studying the effect of cognitive style according to the (FD-I) theory.

### Interaction, Adaptation, and Personalization

As with the study of the effects of individual user differences, there have been extensive studies of novel interaction and adaptation mechanisms outside of the area of Information Visualization. For example, related work has looked at a variety of adaptation techniques, such as display notifications (Bartram et al., 2003), hint provisions (Muir and Conati, 2012), search result reranking (Steichen et al., 2012), or adaptive navigation (Steichen et al., 2012).

In Information Visualization, the most common interaction and adaptation technique has typically been to recommend alternative visualizations (Grawemeyer, 2006; Gotz and Wen, 2009). More recently, Kong et al. developed a system that could dynamically add overlays to a visualization in order to aid chart understanding (Kong and Agrawala, 2012). In particular, the developed overlays were “reference structures” (e.g., grids), “highlights” (e.g., highlighting a particular bar in a bar graph), “redundant encodings” (e.g., data labels), “summary statistics” (e.g., mean line), and “annotations” (e.g., providing comments on particular data points). However, no studies were performed to investigate the relative benefits, drawbacks, or individual user preferences.

Most closely to our work, Carenini et al. (2014) proposed the personalization of visualizations that a user currently engages with [rather than providing personalized recommendations for alternative visualizations as in Grawemeyer (2006) and Gotz and Wen (2009)]. The actual adaptation techniques proposed in Carenini et al. (2014) were inspired by an analysis of classical Infovis literature (Bertin, 1983; Kosslyn, 1994), as well as a seminal taxonomy on “visual prompts” from Mittal (1997). Similar to the abovementioned “overlay techniques” in Kong and Agrawala (2012), these “visual prompts” were a collection of visualization overlays and parameters that could be added or changed on a visualization, either interactively or adaptively. In particular, Carenini et al. (2014) focused on a subset of “visual prompts” from Mittal (1997) that could be used for highlighting specific data points that are relevant to the user's current task. The chosen techniques in Carenini et al. (2014) therefore require a system to have exact knowledge of the user's task, e.g., knowing exactly which two data points on a graph the user is interested in comparing with each other. This assumption is based on the idea of “*Magazine Style Narrative Visualization*” as presented in Segel and Heer (2010) and Kong et al. (2014), where the visualization is meant to accompany a known textual narrative (Segel and Heer, 2010).

However, this assumption of knowing the exact elements of interest to the user cannot be guaranteed for visualizations in general. By contrast, the work in our paper focuses on visual prompts, called “visualization aids” in our paper, that can be added to a visualization without knowing the exact data points that a user is interested in (e.g., reference structures, such as grids), thereby making them task-independent and applicable for different types of scenarios. In addition, our work explores the effect of cognitive style on aid usage and preferences.

## Experimental Setup

In order to study user behaviors and preferences with regards to different interactive visualization aids, as well as the effects of a user's cognitive style, we conducted a laboratory experiment involving two different visualizations, as well as five different visualization aids. Overall, 40 participants took part in the study, which consisted of a series of visualization tasks to be completed using the given visualizations. The following paragraphs describe the visualizations and aids used in the study, the study tasks and procedure, as well as the participant recruitment and data analysis.

### Visualizations and Aids Used in the Study

The study was conducted using two visualization types, namely bar graphs and line graphs. The choice for these visualizations was based on their ubiquitous adoption across different fields and media, as well as some use in prior work on user differences (e.g., bar graphs in Toker et al., 2012).

For each of the visualizations, five visualization aids were available to participants, which were largely based on the “visual prompts” taxonomy presented in Mittal (1997) (and also used in Carenini et al., 2014). In particular, each of these visualization aids fall into the “overlay possible (*ad-hoc*)” category (i.e., aids that can be overlaid dynamically, even by a third-party software as presented in Kong and Agrawala, 2012), as opposed to “planned with original design” (i.e., requiring significant changes to the graph that could only be made if included in advance by the original visualization designers, e.g., axis change, typeface change). As such, they also adhere to the “reference structures” and “redundant encodings” categories from the taxonomy in Kong and Agrawala (2012).

The choice for these particular types of aids was based on the fact that they can be used as an overlay on an existing visualization (and may therefore be used as an interactive or adaptive help for users), and that they do not require any knowledge of the user's focus on any particular data point. In addition, all of the chosen aids were applicable to both bar graphs and line graphs (and potentially other visualizations), thereby also allowing an analysis of any effects of visualization type.

**Figure 1:**
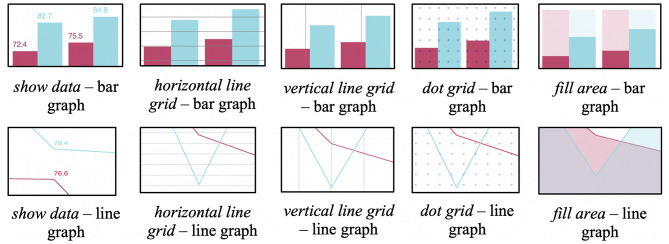
Visualization aids used in the study.

Figure 1 shows all five aids, for both bar graphs and line graphs. Specifically, the aids were:

- *show data*—adding the exact data point values above the respective bar/line. The hypothesis for this aid is that it helps users who have difficulties in comparing two data points using purely graphical representations.
- *horizontal line grid*—overlaying a horizontal grid. The hypothesis for this aid is that it helps users in comparing specific points across a graph through additional structure (e.g., for comparing the height of two bars that may be on opposite sides).
- *vertical line grid*—overlaying a vertical grid. The reason for including this aid in the study is the hypothesis that some participants may like to combine horizontal and vertical lines to form additional structure that may help in dissecting a visualization.
- *dot grid*—overlaying a dot grid. This aid is included as an alternative to the above solid grids, as it may be preferred as a less intrusive option.
- *fill area*—adding a shaded complement in a bar graph/adding a shaded area underneath a line for the line graph. This aid thereby represents an alternative reference structure aid. The hypothesis is that some users may prefer the provided additional visual representations, e.g., some users may always prefer to compare shorter or longer bars, or use the visual cues provided by overlaps in the line graphs.

Each of these aids could be toggled on and off by users through checkboxes. In addition, the system allowed users to toggle multiple aids (i.e., overlay) at any given time. Also, the order of aid checkboxes was randomized on a per-participant basis, to minimize any ordering effects while still maintaining a consistent interface for each individual participant.

### Experimental Tasks

**Figure 2:**
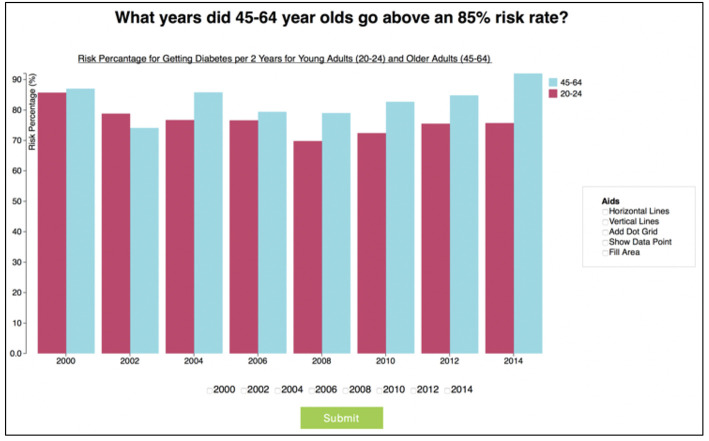
Sample task from the diabetes data set, with low information density bar graph.

Each participant performed a set of tasks related to two standard datasets drawn from Data.gov, namely the Diabetes Data Set^1^ and the Los Angeles Crime ^2^ dataset. A task consisted of a question, a corresponding graph, and a set of possible answers (see Figure 2).

Half of the questions required the choice of only one answer (using radio buttons), with the other half allowing the choice of multiple correct answers (using checkboxes). The tasks were designed to be of varying type and complexity. In particular, the questions were based on the taxonomy of task types presented in Amar et al. (2005), and consisted of “Retrieve Value,” “Compute Derived Value,” “Filter,” and “Find Extremum” tasks.

**Figure 3:**
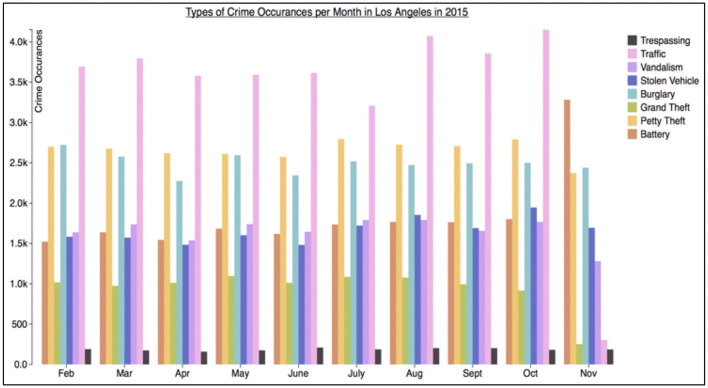
Sample high information density graph for the Los Angeles crime data set.

Furthermore, the graphs were either of “Low Information Density,” which showed only two series (as in Figure 1), or “High Information Density,” which showed seven series (see Figure 3 for an example of a “High Information Density Bar Graph”). This distinction was included to facilitate the analysis of potential effects of information density on aid usage. For example, it may be the case that aids are not considered important for “Low Information Density” graphs, while some/all participants may see a benefit of aids for “High Information Density” graphs.

### Procedure

Each participant followed the same study procedure, which started with the agreement to a consent form. This was followed by demographic questionnaires regarding participant age, gender, as well as self-reported experience/expertise with different types of visualizations. Specifically, they were asked how often they work with high/low information density bar/line graphs, on a scale from 1 (never) to 5 (very frequently).

Each participant was then presented with the same two practice tasks (one per visualization type, each using high information density), where they were encouraged to familiarize themselves with the graph layouts and question/answer types, as well as to try out all of the aids.

Following the practice tasks, participants performed 50 tasks (25 with each visualization; total of 20 high information density, 30 low information density), where graph type, task question, and information density were all counterbalanced across participants to avoid any ordering effects. For each task, the participant's time was recorded, along with all mouse clicks.

After all tasks were completed, participants filled out a post-task questionnaire, where they noted their perceived usefulness of the different aids (on a 5-point Likert scale).

Lastly, users' cognitive styles according to the FD-I theory were measured through the Group Embedded Figures Test (GEFT)^3^ (Oltman and Witkin, 1971), which is a reliable and validated test that has been frequently used in prior research (e.g., Mawad et al., 2015; Raptis et al., 2016).

The average session lasted ~1 h, and each participant was compensated with a $20 gift voucher.

### Participant Recruitment and Demographics

40 participants were recruited by the authors through University mailing lists. The age range was between 18 and 77 (average of 28 years), 24 participants were female, and 16 were male. The participants consisted of students, faculty, and administrators. There was a balanced distribution across colleges and departments (e.g., arts, business, engineering, science), thereby ensuring minimized bias toward any domain-specific population. The average GEFT score was 13.75/18 (SD = 4.24), suggesting the population was slightly biased toward field independence. The average self-rated expertise of participants was 3.18 (SD = 0.93) out of 5 for “Simple Bar” visualizations, 2.50 (SD = 1.04) for “Complex Bar” visualizations, 3.40 (SD = 0.87) for Simple Line visualizations, and 2.80 (SD = 0.88) for Complex Line visualizations.

### Data Analysis

All data was analyzed using General Linear Models (GLM), which are a generalization of ordinary linear regression models (i.e., a generalization that incorporates a number of different statistical models, such as ANOVA, ANCOVA, MANOVA, MANCOVA, ordinary linear regression, *t*-test, and *F*-test) (Field, 2009). The independent measures used in the models were graph type, information density, user cognitive style, and user expertise. The dependent measures were accuracy (whether participants submitted the correct answer), task time (measured from the start of a task to pressing the submit button), aid count (how frequently participants made use of specific aids), and subjective preferences (from the post-task questionnaire).

## Results

This section presents the general results for each of the dependent measures, i.e., accuracy, time, aid count, and preferences. In addition, this section reports on our analysis of the influence of a user's cognitive style on these measures. Expertise (as measured through the self-reported questionnaire) did not have an effect on any of the measures and is therefore not reported further.

### Accuracy

Overall, the mean accuracy across all participants was very high at 87% (43.72 correct tasks out of 50). It therefore appears that participants may have been taking as much time as needed to get the correct answer, i.e., they may have been penalizing time for accuracy (similar to results found in Toker et al. (2012) and Carenini et al. (2014). No effects were found for any of the independent factors on this measure, most likely because of the overall high accuracy (and therefore lack of variance) across participants.

### Time

Participants took on average 29.75 s to complete a task, with a standard deviation of 19.47 s. As expected, high information density tasks took considerably longer (34.76 s) compared to low information density tasks (23.56), and this difference was statistically significant (*F*_1,39_ = 44.31, *p* < 0.001). Likewise, graph type played a small role, with participants taking slightly longer with Bar graphs (30.30 s) compared to Line graphs (28.03). This difference was also statistically significant (*F*_1,39_ = 4.07, *p* < 0.05). In addition, there was a statistically significant (*F*_1,39_ = 10.583, *p* < 0.05) interaction effect between graph type and information density, with high information density tasks showing a difference between the two graphs, while both graphs performed almost equally on low density tasks.

**Figure 4:**
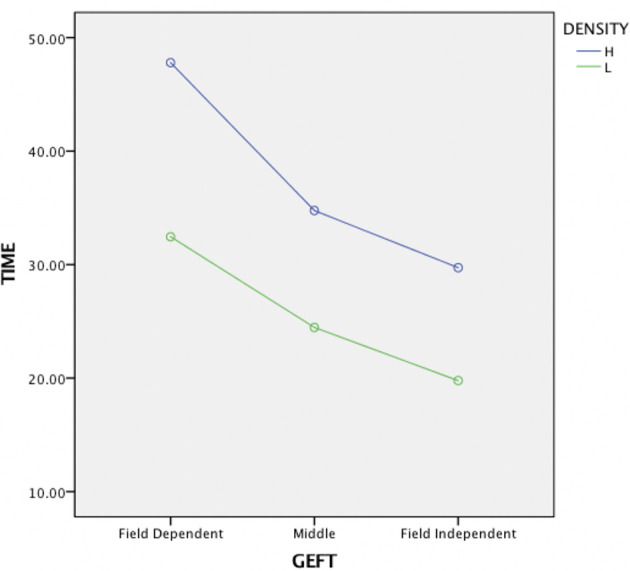
Effects of cognitive style (GEFT) and Information density on task time.

A user's cognitive style, as measured through the GEFT, had a statistically significant effect on a user's time on task. Specifically, participants with high field independence scores had statistically significantly faster times than participants with low field independence scores (*F*_1,39_ = 187.60, *p* < 0.001). When using a three-way split [as recommended by Cureton (1957)], which differentiates between Field Independent (FI-upper 27%), Field Dependent (FD-lower 27%), and Middle participants, FD participants (*N* = 10) were found to take 36.5 s, Middle participants (*N* = 19) 27 s, and FI participants (*N* = 11) 23.5 s (see Figure 4). This finding was slightly more pronounced for high density tasks compared to low density tasks (*F*_1,39_ = 6.70, *p* < 0.01). Lastly, aid use did not lead to any statistically significant performance increases for FD or FI participants.

### Aid Count

**Figure 5:**
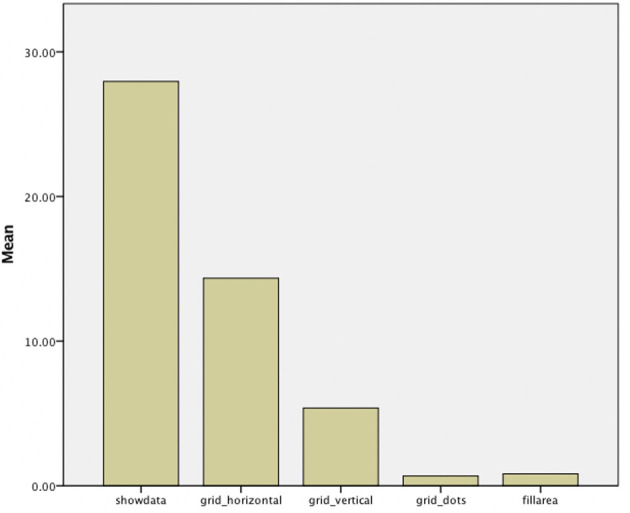
Average use of aids per participant (across 50 tasks).

Overall, participants turned on an aid 1,967 times (average of 49.17 per participant). The most popular aids were *show data* (27.95 uses on average) and *horizontal line grid* (14.35), while the other aids were less popular, with 5.3 uses for *vertical grid line* and only 0.67 and 0.82 uses for *dot grid* and *fill area*, respectively (see Figure 5). This difference between aids was statistically significant (*F*_4,39_ = 32.22, *p* < 0.001).

This general trend was found across both graphs. Additionally, there was a statistically significant effect of graph type on the use of the *vertical line grid* (*F*_1,39_ = 5.23, *p* < 0.03), with this aid being more popular for the line graph (average of 5.37 uses per participant) compared to the bar graph (average of 1.17 uses per participant). Task Density, however, showed no statistically significant effect overall on any aid counts.

**Figure 6:**
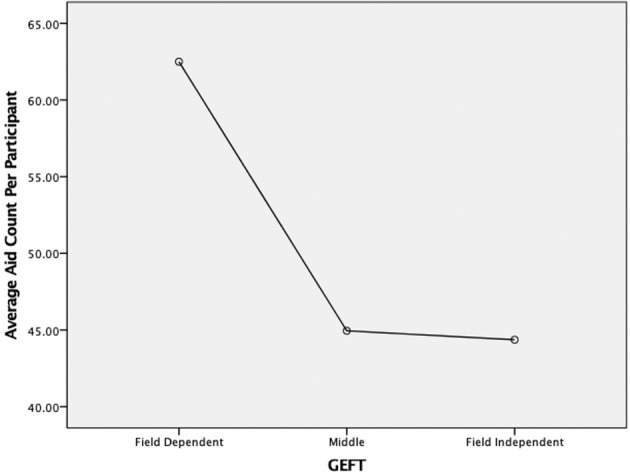
Effect of cognitive style on aid count—overall.

**Figure 7:**
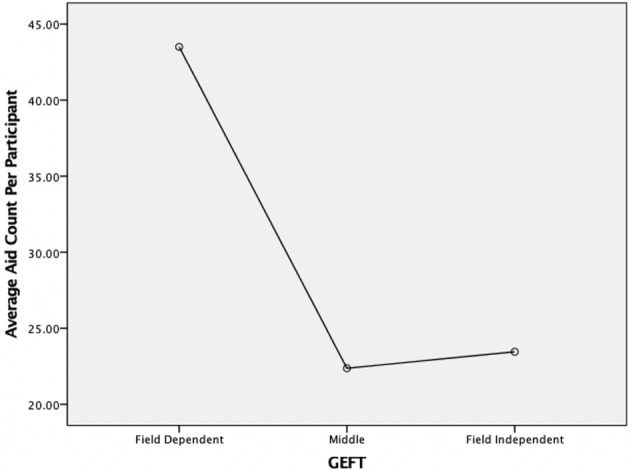
Effect of cognitive style on aid count—s*how data* aid only.

A participant's cognitive style had a statistically significant effect on aid count, with FD participants making substantially more use of aids compared to FI participants (*F*_1,39_ = 7.25, *p* < 0.01). Specifically, when using a three-way split, FD participants were found to use 63.5 aids on average, Middle participants used 46 aids, and FI participants used 45.36 aids (see Figure 6). When further breaking down these results, it was found that the difference between FD and Middle/FI participants was particularly striking for the *show data* aid. Specifically, FD participants used *show data* aids 43.5 times, vs. only 22.36 times for middle and 23.45 times for FI participants (see Figure 7). This result was found to be statistically significant (*F*_1,39_ = 6.81, *p* < 0.003). Aid use for the other aids was almost equal between FD and FI participants, and, in fact, FI users chose *horizontal grid* slightly more often than FD users (14.9 vs. 13.9, ns). Task Density only had a marginally significant interaction effect with cognitive style (*F*_1,39_ = 1.85, *p* < 0.055), with FD participants having a slightly more elevated use of aids during high density tasks.

### Preferences

**Figure 8:**
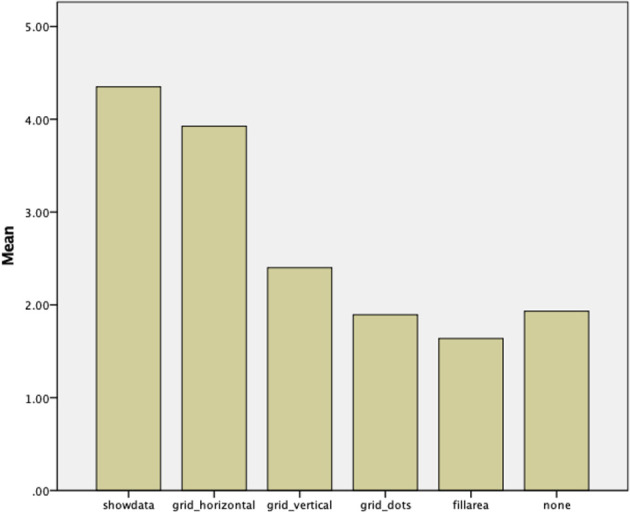
Perceived aid usefulness (5 = very useful, 1 = not useful at all), “none” refers to the usefulness of not having any aids overlaid.

The analysis of participants' subjective preferences for the different aids (from the post-task questionnaire) revealed similar results to the aid count analysis above. In particular, both *show data* and *horizontal line grid* were considered the most useful, with usefulness scores of 4.35 and 3.93, respectively (5 being very useful, and 1 not being useful at all), while none of the other aids (or *no aid*) were considered useful (2.4 for *vertical line grid*, 1.9 for *dot grid*, 1.7 for *fill area*, and 2.0 for *no aid*) (see Figure 8). This difference between aids was again statistically significant (*F*_5, 39_ = 77.36, *p* < 0.001).

Likewise, there was a statistically significant effect of graph type for the *vertical line grid* (*F*_1,39_ = 100.56, *p* < 0.001), with this aid being considered more useful for the line graph (3.26) compared to the bar graph (1.54).

As with aid count, there was a statistically significant effect of cognitive style on perceived usefulness, with FD participants reporting greater usefulness across all aids overall. Again, *show data* in particular showed a statistically significant effect, with a rating of 4.6 for FD participants, 4.35 for Middle, and 4.1 for FI participants (*F*_1,39_ = 4.77, *p* < 0.03).

## Summary and Discussion

The study has revealed a number of interesting findings regarding the three research questions posed in the introduction. This section first provides a summary of these findings, as well as implications for design and potential adaptation, followed by a discussion of the limitations of the study.

Firstly, the results from the study have shown that cognitive style indeed plays a significant role when performing tasks with Information Visualizations (RQ1). In particular, FI users have been shown to be significantly faster at completing tasks compared to FD users. This is in line with similar work in Information Visualization regarding other cognitive measures, such as perceptual speed or working memory (e.g., Velez et al., 2005; Toker et al., 2012; Carenini et al., 2014). Similar to design suggestions in such prior work, this may indicate that FD participants may be in particular need to receive additional help in performing visualization tasks, such as through adaptive or personalized aid additions or recommendations. In contrast to task time, however, the study did not reveal any differences in accuracy, possibly due to participants sacrificing task time for accuracy (again, in line with prior work on other user characteristics (e.g., Toker et al., 2012; Carenini et al., 2014).

In general, there were also clear differences in terms of visualization aid choices and preferences (RQ2), with *show data* and *horizontal grid lines* being most used and subjectively preferred. When designing interactive visualizations with visualization aids, it may therefore be advisable to use one of these two options (or both). The fact that *vertical grid lines* were less used and preferred is understandable (especially for the bar graph), given that they provide less support in judging data point values (since they only produce additional partitioning of the graph). As previously mentioned, the reason for including this in the study was the hypothesis that some participants may like to combine horizontal and vertical lines to form additional structure that may help in dissecting a visualization. However, this seems not to have been the case. The low use of *fill area* or *dot grid* is less clear, although it is conceivable that, at least for the *fill area* aid, high information density tasks may have suffered too much of a performance decrease (due to the many overlaps in line graphs, and the high number of different colors/shades for bar graphs). However, *post-hoc* analyses did not find an effect for information density on this particular aid. The *dot grid* area had been included in the study as an alternative to the solid grids, as it may have been preferred as a less intrusive option. However, it appears that it was not judged to be useful. This suggests that, if grids are to be added, they should be solid lines in order to provide better support for users.

Cognitive style was also shown to play an important role in visualization aid choice and subjective usefulness (RQ3), with FD users making significantly more use of visualization aids during their tasks. Likewise, they clearly noted them more as being useful in completing the tasks, as shown through the final survey. While our study was not able to detect a performance increase when using aids, the fact that participants continued to choose them throughout the study suggests that they felt a benefit, even if just in terms of subjective experience. This suggests that there may be implications of cognitive style to information visualization design, with FD users potentially benefitting most from a system that perhaps adds these aids by default, or one that adds them adaptively (or provides them as recommendations). In terms of specific aid choices, it was shown that FD participants made significant use of the *show data* aid (i.e., the aid that overlays the actual data values), and that they strongly considered this aid to be useful. This suggests that FD participants may have more difficulties using purely visual representations of data, and that they prefer to have additional numerical data displayed in the visualization. This is in line with the general definitions of cognitive style along the FD-I dimension, i.e., FD participants struggling to identify details in complex visual scenes. *Grid horizontal* was also chosen by FD participants to a certain degree, but the fact that it was used significantly less suggests that the added structure through additional visual objects was not appreciated as much by such users. As with research on other forms of cognitive style (e.g., along the verbalizer-visualizer dimension), this may again suggest that additional (non-visual) forms of cognitive aids should be explored for FD users. FI users chose aids significantly less often overall (particularly the *show data* aid), which may potentially suggest that such users might prefer the option to interactively turn on aids themselves, rather than systems where aids are turned on by default. However, this hypothesis requires further research (discussed below).

Lastly, there are a number of limitations of the study, some of which may be addressed in future work (also discussed in the following section). First of all, the study consisted of a laboratory study with 40 participants, 2 visualizations, and 5 visualization aids. While the number of participants was in line with similar prior work (e.g., Bartram et al., 2003; Velez et al., 2005; Grawemeyer, 2006; Green and Fisher, 2010; Toker et al., 2012; Carenini et al., 2014; Raptis et al., 2016) and provided sufficient strength to reach statistical significance for several effects, a larger participant pool may have enabled the discovery of additional effects, as well as the study of a larger set of visualization types and aids. Specifically, there are many other basic visualizations and aids that could be studied, including the various types of visualizations and aids proposed in Kong and Agrawala (2012). While the purpose of our study was to focus on particularly common visualizations (bar and line graphs) and aids (e.g., grids, labels, etc.), such future investigations of additional basic visualizations could thereby potentially identify which specific (aspects of) visualizations would most benefit from providing aids to users. Likewise, there are many more complex and/or domain-specific visualizations, aids, and tasks that could be studied (e.g., specific visualizations and tasks for decision-making, such as in Conati et al., 2014). While the focus of our study was on basic, common, and domain-independent visualizations and tasks, more complex visualizations, aids, and tasks could potentially bring out even stronger results. Specifically, complex visualizations may elicit more aid usage from users, and also potentially reveal even bigger differences depending on cognitive style.

## Conclusions and Future Work

Overall, the results from the study provide valuable information regarding the role of user cognitive style in Information Visualization, as well as initial implications for the design of Information Visualization aids. In particular, it was shown that cognitive style has a significant impact on visualization task performance, that different visualization aids are chosen to different degrees, and that cognitive style has a significant influence on which aids are chosen and considered most helpful.

The paper thereby provides further motivation for the development of adaptive and personalized Information Visualization systems, as previously proposed in related work (e.g., Grawemeyer, 2006; Gotz and Wen, 2009; Toker et al., 2012; Carenini et al., 2014; Steichen et al., 2014). In particular, the paper provides the first results that motivate the adaptation and personalization of Information Visualization systems depending on a user's cognitive style, through the use of visualization aids.

Based on this motivation, there are several avenues for further research. As discussed in the previous section, there are many additional visualizations and aids that may be studied, which may uncover whether there are particular (aspects of) visualizations that elicit differences depending on cognitive style. Such studies of larger sets of visualizations would require the recruitment of larger participant pools, in order to ensure the same statistical power. In addition, the study of more complex and/or domain-specific visualizations and tasks may provide further insights into the role of cognitive style on different (types of) visualizations, and/or potentially reveal specific application scenarios where aids may be particularly useful.

Furthermore, additional research needs to be conducted in order to study the effects of adding visual aids by default, or adding them adaptively while a user is performing a task (e.g., as in Carenini et al., 2014). While our study did not find a significant performance improvement from aid usage (potentially due to participants needing to spend time to choose and turn on aids), such research may also be able to better quantify such effects. These findings would complement the aid choice and perceived usefulness results from this paper. Since *show data* and *horizontal grid* were by far the most popular aids in our current study, an initial investigation of default/adaptive aid addition may specifically focus on these two visualizations (as well as perhaps additional non-visual aids for FD users).

Furthermore, in order to develop a personalized system, an adaptive aid component would also need to be integrated with a system that can automatically recognize a user's cognitive style, for example using eye gaze data (as in Raptis et al., 2017). Our future research will involve the development of such systems, by extending work in Raptis et al. (2017) to the field of Information Visualization. Given the successful results in Raptis et al. (2017), as well as successful predictions (using eye gaze) of other user types of user characteristics during visualization tasks (e.g., perceptual speed and working memory in Steichen et al., 2014), we hypothesize that this is an achievable task.

Finally, once all components have been developed and evaluated, the final stage of research will involve an investigation of integrated systems that adaptively add or suggest aids based on a user's predicted cognitive style.

## Data Availability Statement

The datasets generated for this study will not be made publicly available.

## Ethics Statement

The studies involving human participants were reviewed and approved by the Human Subjects Committee of the primary author's institution. The participants provided their written informed consent to participate in this study.

## Author Contributions

Both authors listed have made a substantial, direct, and intellectual contribution to the work, and approved it for publication.

### Conflict of Interest

The authors declare that the research was conducted in the absence of any commercial or financial relationships that could be construed as a potential conflict of interest.

## Footnotes

^1^https://catalog.data.gov/dataset/diabetes

^2^https://catalog.data.gov/dataset/crime-data-from-2010-to-present-c7a76

^3^For this study, we used the online version of the test—https://www.mindgarden.com/105-group-embedded-figures-test-a-measure-of-cognitive-style
